# Supplementary material for: Analyzing enterprise asset structure and profitability using cloud computing and strategic management accounting
Source: PLoS One. 2021 Sep 30;16(9):e0257826. doi: 10.1371/journal.pone.0257826 (PMC8483362; doi:10.1371/journal.pone.0257826)
Supplement: Attachment — Submitted filename: Response to Reviewers.docx [file pone.0257826.s002.docx]

| **Date:** | Jun 14 2021 02:31PM |
| --- | --- |
| **To:** | "Wenquan Shi" wqshi@szai.edu.cn |
| **From:** | "PLOS ONE" plosone@plos.org |
| **Subject:** | PLOS ONE Decision: Revision required [PONE-D-21-06810] |

PONE-D-21-06810
Analyzing Enterprise Asset Structure and Profitability Using Cloud Computing and Strategic Management Accounting
PLOS ONE

Dear Dr. Shi,

Thank you for submitting your manuscript to PLOS ONE. After careful consideration,I feel that it has merit but does not fully meet PLOS ONE’s publication criteria as it currently stands.

Both reviewers consider that there is a lack of motivation in this research. I cannot guarantee that after the revision the paper will be suitable of publication considering the concerns showed by one of the reviewers regarding to the methodology used, specifically, with the dada considered.

Authors must provide a reasonable explanation of these major concerns.

Other minor questions are that paper should include more discussion engaging in the dialogue with the relevant literature as well as the future perspectives and limitations of this research. Therefore, I invite you to submit a revised version of the manuscript that addresses the points raised during the review process.

Please submit your revised manuscript by Jul 29 2021 11:59PM. If you will need more time than this to complete your revisions, please reply to this message or contact the journal office at plosone@plos.org. When you're ready to submit your revision, log on to https://www.editorialmanager.com/pone/ and select the 'Submissions Needing Revision' folder to locate your manuscript file.

Please include the following items when submitting your revised manuscript:

- A rebuttal letter that responds to each point raised by the academic editor and reviewer(s). You should upload this letter as a separate file labeled 'Response to Reviewers'.
- A marked-up copy of your manuscript that highlights changes made to the original version. You should upload this as a separate file labeled 'Revised Manuscript with Track Changes'.
- An unmarked version of your revised paper without tracked changes. You should upload this as a separate file labeled 'Manuscript'.

If you would like to make changes to your financial disclosure, please include your updated statement in your cover letter. Guidelines for resubmitting your figure files are available below the reviewer comments at the end of this letter.

If applicable, we recommend that you deposit your laboratory protocols in protocols.io to enhance the reproducibility of your results. Protocols.io assigns your protocol its own identifier (DOI) so that it can be cited independently in the future. For instructions see: [http://journals.plos.org/plosone/s/submission-guidelines#loc-laboratory-protocols](http://journals.plos.org/plosone/s/submission-guidelines#loc-laboratory-protocols). Additionally, PLOS ONE offers an option for publishing peer-reviewed Lab Protocol articles, which describe protocols hosted on protocols.io. Read more information on sharing protocols at <https://plos.org/protocols?utm_medium=editorial-email&utm_source=authorletters&utm_campaign=protocols>.

We look forward to receiving your revised manuscript.

Kind regards,

J E. Trinidad Segovia
Academic Editor
PLOS ONE

Journal Requirements:

When submitting your revision, we need you to address these additional requirements.

1. Please ensure that your manuscript meets PLOS ONE's style requirements, including those for file naming. The PLOS ONE style templates can be found at

https://journals.plos.org/plosone/s/file?id=wjVg/PLOSOne_formatting_sample_main_body.pdf and

[https://journals.plos.org/plosone/s/file?id=ba62/PLOSOne_formatting_sample_title_authors_affiliations.pdf](https://www.editorialmanager.com/pone/about:blank)

2. PLOS requires an ORCID iD for the corresponding author in Editorial Manager on papers submitted after December 6th, 2016. Please ensure that you have an ORCID iD and that it is validated in Editorial Manager. To do this, go to ‘Update my Information’ (in the upper left-hand corner of the main menu), and click on the Fetch/Validate link next to the ORCID field. This will take you to the ORCID site and allow you to create a new iD or authenticate a pre-existing iD in Editorial Manager. Please see the following video for instructions on linking an ORCID iD to your Editorial Manager account: https://www.youtube.com/watch?v=_xcclfuvtxQ

3.  Please include captions for your Supporting Information files at the end of your manuscript, and update any in-text citations to match accordingly. Please see our Supporting Information guidelines for more information: [http://journals.plos.org/plosone/s/supporting-information](https://www.editorialmanager.com/pone/about:blank).

[Note: HTML markup is below. Please do not edit.]

Reviewers' comments:

Reviewer's Responses to Questions

**Comments to the Author**

1. Is the manuscript technically sound, and do the data support the conclusions?

The manuscript must describe a technically sound piece of scientific research with data that supports the conclusions. Experiments must have been conducted rigorously, with appropriate controls, replication, and sample sizes. The conclusions must be drawn appropriately based on the data presented.

Reviewer #1: Yes

Reviewer #2: Partly

2. Has the statistical analysis been performed appropriately and rigorously?

Reviewer #1: Yes

Reviewer #2: No

3. Have the authors made all data underlying the findings in their manuscript fully available?

The [PLOS Data policy](http://www.plosone.org/static/policies.action#sharing) requires authors to make all data underlying the findings described in their manuscript fully available without restriction, with rare exception (please refer to the Data Availability Statement in the manuscript PDF file). The data should be provided as part of the manuscript or its supporting information, or deposited to a public repository. For example, in addition to summary statistics, the data points behind means, medians and variance measures should be available. If there are restrictions on publicly sharing data—e.g. participant privacy or use of data from a third party—those must be specified.

Reviewer #1: Yes

Reviewer #2: No

4. Is the manuscript presented in an intelligible fashion and written in standard English?

PLOS ONE does not copyedit accepted manuscripts, so the language in submitted articles must be clear, correct, and unambiguous. Any typographical or grammatical errors should be corrected at revision, so please note any specific errors here.

Reviewer #1: Yes

Reviewer #2: No

5. Review Comments to the Author

Please use the space provided to explain your answers to the questions above. You may also include additional comments for the author, including concerns about dual publication, research ethics, or publication ethics. (Please upload your review as an attachment if it exceeds 20,000 characters)

Reviewer #1: This research is mainly for the financial analysis of express delivery companies, mainly explores the financial indicators and non-financial indicators of express delivery companies, and analyzes the factors that affect the sustainable profitability of the express delivery industry. At the same time, the article uses the current advanced cloud computing data processing methods to analyze the market competitiveness and commercial value of express delivery companies, and conducts a more comprehensive and scientific analysis for the express delivery companies studied. I think the research and analysis method of this article is reasonable and scientific, but I think this article can be published after modification.

The main problems of this article are as follows:

1. It is recommended that authors include specific values in the abstract, as this can improve the scientific nature of the article. It is recommended that the authors modify this.

Response: thank you for your valuable advice! We have included certain values in the abstract to demonstrate the scientific nature of our work.
2. In the introduction, the author stated that "In 2019, express service enterprises’ business volume reached 50.71 billion pieces, with a year-on-year increase of 26.6%." The author needs to explain the source of this data.

Response: thank you for your valuable suggestions! We have added references to the relevant data in the introduction to illustrate this phenomenon.
3. The last paragraph in the introduction does not need to explain all the research content, only the main purpose and method of the research need to be explained. I suggest the author to delete this part.

Response: thank you for your valuable opinion! We have supplemented the description of the main objectives and methods of the present study in the last paragraph of the introduction as required.
4. In terms of literature review, the author needs to explain the previous research results, and the limitations of the previous research and the improvements that need to be made.

Response: thank you for your valuable advice! We have added the limitations of the current research and the areas for improvement in the literature review “A Summary of Previous Works”, according to your suggestions.
5. The enterprise analysis model is the key content of the research, and the author needs to explain the model in Figure 1 more completely.

Response: thank you for your valuable advice! We have supplemented the detail explanation of the current model in Figure 1 as suggested.
6. The author needs to explain the specific parameters and steps of cloud computing data processing used in the research.

Response: thank you for your valuable advice! We have added the specific parameters and corresponding steps for cloud computing data processing.
7. It would be more appropriate to change the "Strategic management accounting analysis" section of the article to "research data sources".

Response: thank you for your valuable comment! We have changed the strategic management accounting analysis to research data sources as required.
8. What is the experimental environment for cloud computing processing used? please explain carefully.

Response: thank you for your valuable opinions! We have supplemented the detail description of the experimental environment for cloud computing processing.
9. The conclusion is an explanation of the research results of the article, so the author needs to explain the specific results of the research in the conclusion.

Response: thank you for your valuable advice! We have included specific numerical values in our conclusions to demonstrate the reliability of our research.

Reviewer #2: 1. The authors had better rewrite the abstract section to appeal to readers. In other words, authors have to explain why your findings matter as well as why your work would contribute to the existing literature.
Response: thank you for your valuable suggestions! We have revised the abstract of the paper and illustrated the key findings of the current study and the major contributions to the current field.
2. Readers prefer finding the strong motivation, purpose, importance of this study, and even the contribution of this study in the introduction section.
Response: thank you for your valuable opinions! We have re-stated the main motivation, purpose and innovation of this research in the introduction.
3. Selecting total assets, current assets, and intangible assets to analyze the enterprise asset structure might be reasonable; however, accounts payable might not be appropriately selected to analyze asset structure due to account payable is not an asset item in the balance sheet.
Response: thank you for your valuable advice! Considering that accounts payable is also a key index in financial analysis, which determines the liquidity performance of the company's assets, so we finally choose this index for analysis.
4. Why do authors select debt repayment, operation, profitability, and growth capabilities to analyze enterprise profitability? Authors have to provide supporting arguments for these items except profitability.
Response: thank you for your valuable suggestion! We have added relevant references in the method section to illustrate that the selection of relevant indicators for the evaluation of corporate profitability is scientific.
5. Reviewers prefer statistical results with enough samples instead of trend charts, pie charts, etc. since the latter (i.e., trend charts, pie charts, etc.) would be difficult to persuade readers. In addition, Figures 1-11 are not shown in the charts shown at the bottom of this paper, which might not be welcomed by reviewers.
Response: thank you for your valuable comments! The Figure 1-11 was provided to the Editorial Department in the form of attachment, which is the journal requirement. We have added the corresponding chart content in the article, and diversified our statistical results displayed in various forms.
6. Providing and comparing descriptive statistics of relevant asset items for different years and different companies as your revealed findings might not be welcomed by academic journals.
Response: thank you for your valuable advice! We have excluded the descriptive statistical results of different company asset items in different years.
7. In addition to implication sections, this paper should include more discussion engaging in the dialogue with the relevant literature.
Response: thank you for your valuable opinions! We have included the content of discussion and compared the results of this paper with previous studies to further verify the effectiveness of the model proposed in this paper.
8. Proposing future perspectives for the study should be mentioned specifically; in addition, authors have to tell readers why such future perspectives are worthwhile for further research as well.

Response: thank you for your valuable suggestions! We have rewritten the conclusion of the paper and detailed the need for future research.

6. PLOS authors have the option to publish the peer review history of their article ([what does this mean?](https://journals.plos.org/plosone/s/editorial-and-peer-review-process#loc-peer-review-history)). If published, this will include your full peer review and any attached files.


If you choose “no”, your identity will remain anonymous but your review may still be made public.


**Do you want your identity to be public for this peer review?** For information about this choice, including consent withdrawal, please see our [Privacy Policy](https://www.plos.org/privacy-policy).

Reviewer #1: **Yes:** Xin Gao

Reviewer #2: No

[NOTE: If reviewer comments were submitted as an attachment file, they will be attached to this email and accessible via the submission site. Please log into your account, locate the manuscript record, and check for the action link "View Attachments". If this link does not appear, there are no attachment files.]

While revising your submission, please upload your figure files to the Preflight Analysis and Conversion Engine (PACE) digital diagnostic tool, <https://pacev2.apexcovantage.com/>. PACE helps ensure that figures meet PLOS requirements. To use PACE, you must first register as a user. Registration is free. Then, login and navigate to the UPLOAD tab, where you will find detailed instructions on how to use the tool. If you encounter any issues or have any questions when using PACE, please email PLOS at [figures@plos.org](mailto:figures@plos.org). Please note that Supporting Information files do not need this step.

*In compliance with data protection regulations, you may request that we remove your personal registration details at any time. [(Remove my information/details)](https://www.editorialmanager.com/pone/login.asp?a=r). Please contact the publication office if you have any questions.*
