# Supplementary material for: Analyzing enterprise asset structure and profitability using cloud computing and strategic management accounting
Source: PLoS One. 2021 Sep 30;16(9):e0257826. doi: 10.1371/journal.pone.0257826 (PMC8483362; doi:10.1371/journal.pone.0257826)
Supplement: Attachment — Submitted filename: Comments for PLOS One 0822-10.docx [file pone.0257826.s003.docx]

**Referee report on manuscript PONE-D-21-06810R1:**

**"Analyzing Enterprise Asset Structure and Profitability Using Cloud Computing and Strategic Management Accounting"**

After reading this revision, there are still several issues listed below.

**Comments**

1. The author should shorten your abstract and highlight your concern in this section since the abstract with many contexts might not easily appeal to readers. For example, the author might explain the revealed results with supporting arguments instead of listing statistical numbers (i.e., 51.8%, 45%, etc.) in detail for each finding.

2. We suggest that you had better tell readers the outlay of this paper at the end of the introduction section.
3. According to the format of PLoS One, the authors should not cite references as presented below, and readers can not find these citations in the Reference section.

4. The literature section should include the relevant studies rather than current studies only. We then suggest that the subtitle of this section might not name “Current research…..”, but we agree that the author could propose current research progress in the context of this section.
5. It might not be appropriate that there is only one long paragraph in the Conclusion section. In addition, the author should use “First, Second……” instead of “First, Secondly……” in this section.
